# On $(p,q)$-Szász-Mirakyan operators and their approximation properties

**DOI:** 10.1186/s13660-017-1467-z

**Published:** 2017-08-24

**Authors:** M Mursaleen, AAH Al-Abied, Abdullah Alotaibi

**Affiliations:** 1 Department of Mathematics, Aligarh Muslim University, Aligarh, 202002 India; 2 Operator Theory and Applications Research Group, Department of Mathematics, Faculty of Science, King Abdulaziz University, P.O. Box 80203, Jeddah, 21589 Saudi Arabia

**Keywords:** 41A10, 41A25, 41A36, *q*-integers, $(p, q)$-integers, *q*-Szász-Mirakyan operators, $(p, q)$-Szász-Mirakyan operators, weighted approximation, Voronovskaya theorem

## Abstract

In the present paper, we introduce a new modification of Szász-Mirakyan operators based on $(p, q)$-integers and investigate their approximation properties. We obtain weighted approximation and Voronovskaya-type theorem for new operators.

## Introduction and preliminaries

In the last two decades, there has been intensive research on the approximation of functions by positive linear operators introduced by using *q*-calculus. Lupas [1] was the first who used *q*-calculus to define *q*-Bernstein polynomials, and later Phillips [2] proposed a generalization of Bernstein polynomials based on *q*-integers. Very recently, Mursaleen et al. applied $(p,q)$-calculus in approximation theory and introduced the first $(p,q)$-analogue of Bernstein operators [3]. They investigated the uniform convergence and convergence rate of the operators and also obtained a Voronovskaya-type theorem. Also, $(p,q)$-analogues of Bernstein-Stancu operators [4], Bleimann-Butzer-Hahn operators [5], and Bernstein-Schurer operarors [6] were defined and their approximation properties were investigated. Most recently, the $(p,q)$-analogues of some more operators were defined and their approximation properties were studied in [7–17], and [18]. In this paper, we introduce a $(p,q)$-analogue of Szász-Mirakyan operators. Let us recall some notation and definitions of $(p,q)$-calculus. Let $0< q< p\leq1$. For nonnegative integers *k* and *n* such that $n\geq k\geq0$, the $(p,q)$-integer, $(p,q)$-factorial, and $(p,q)$-binomial are respectively defined by

$$ \begin{gathered} {}[ k]_{p,q}:=\frac{p^{k}-q^{k}}{p-q}, \\ {}[ k]_{p,q}!:=\left \{ \textstyle\begin{array}{l@{\quad}l} {}[ k]_{p,q}[k-1]_{p,q}\cdots1,& k\geq1, \\ 1, & k=0 ,\end{array}\displaystyle \right . \end{gathered} $$

and

$$ \left [ \textstyle\begin{array}{c} n \\ k\end{array}\displaystyle \right ] _{p,q}:= \frac{[n]_{p,q}!}{[k]_{p,q}![n-k]_{p,q}!}. $$

In the case of $p=1$, these notations reduce to *q*-analogues, and we can easily see that $[n]_{p,q}=p^{n-1}[n]_{q/p}$. Further, the $(p,q)$-power basis is defined by

$$ (x\oplus a)_{p,q}^{n}:=(x+a) (px+qa) \bigl(p^{2}x+q^{2}a \bigr)\cdots \bigl(p^{n-1}x+q^{n-1}a \bigr) $$

and

$$ (x\ominus a)_{p,q}^{n}:=(x-a) (px-qa) \bigl(p^{2}x-q^{2}a \bigr)\cdots \bigl(p^{n-1}x-q^{n-1}a \bigr). $$

Also the $(p,q)$-derivative of a function *f*, denoted by $D_{p,q}f$, is defined by

$$ (D_{p,q}f) (x):=\frac{f(px)-f(qx)}{(p-q)x},\quad x\neq 0,\qquad(D_{p,q}f) (0):=f^{{\prime}}(0) $$

provided that *f* is differentiable at 0. The formula for the $(p,q)$-derivative of a product is

$$ D_{p,q} \bigl(u(x)v(x) \bigr):=D_{p,q} \bigl(u(x) \bigr)v(qx)+D_{p,q} \bigl(v(x) \bigr)u(qx). $$

For more details on $(p,q)$-calculus, we refer the readers to [19, 20] and the references therein. There are two $(p,q)$-analogues of the exponential function:

1.1 $$ e_{p,q}(x)=\sum_{n=0}^{\infty} \frac{p^{\frac{n(n-1)}{2}}x^{n}}{[n]_{p,q}!} $$

and

$$ E_{p,q}(x)=\sum_{n=0}^{\infty} \frac{q^{\frac{n(n-1)}{2}}x^{n}}{[n]_{p,q}!} $$

which satisfy the equality $e_{p,q}(x)E_{p,q}(-x)=1$. For $p=1$, $e_{p,q}(x)$ and $E_{p,q}(x)$ reduce to the *q*-exponential functions. Here we note that the interval of convergence of $e_{p,q}(x)$ is $| x|<1/(p-q)$ for $| p|<1$ and $| q|<1$, and series (1.1) converges for all $x\in\mathbb{R}$, $| p|<1$, and $| q|<1$.

## Construction of operators and auxiliary results

We first define the analogue of Szász-Mirakyan operators via $(p, q)$-calculus as follows.

### Definition 2.1

Let $0< q< p\leq1$ and $n\in\mathbb{N}$. For $f:[0,\infty)\rightarrow\mathbb{R}$, we define the $(p, q)$-analogue of Szász-Mirakyan operators by

2.1 $$ {S}_{n,p,q}(f;x)= \sum_{k=0}^{\infty} \frac{p^{ \frac{k(k-1)}{2}}}{q^{ \frac{k(k-1)}{2}}}\frac{([n]_{p,q}x)^{k} }{ [k]_{p,q}! }e_{p,q} \bigl(-[n]_{p,q}q ^{-k}x \bigr) f \biggl(\frac {[k]_{p,q}}{p^{k-1}[n]_{p,q}} \biggr). $$

Operators (2.1) are linear and positive. For $p=1$, they turn out to be the *q*-Szász-Mirakyan operators defined in [21].

### Lemma 2.1

*Let* $0< q< p\leq1$ *and* $n\in\mathbb{N}$. *We have*

2.2 $$ {S}_{n,p,q} \bigl(t^{m+1};x \bigr)= \sum _{j=0}^{m} \left ( \textstyle\begin{array}{c} m \\ j\end{array}\displaystyle \right ) \frac{q^{j}x }{p^{j} [n]_{p,q}^{m-j} }{S}_{n,p,q} \bigl(t^{j};q^{-1}x \bigr). $$

### Proof

Using the identity

$$ [k+1]_{p,q}=p^{k}+q[k]_{p,q}, $$

we can write

$$ \begin{aligned} {S}_{n,p,q} \bigl(t^{m+1};x \bigr) ={}&\sum _{k=0}^{\infty} \frac{p^{ \frac{k(k-1)}{2}}}{q^{ \frac{k(k-1)}{2}}} \frac {([n]_{p,q}x)^{k} }{ [k]_{p,q}! } \biggl(\frac{[k]_{p,q}}{p^{k-1}[n]_{p,q}} \biggr)^{m+1} e_{p,q} \bigl(-[n]_{p,q}q ^{-k}x \bigr) \\ ={}& \sum_{k=0}^{\infty} \frac{p^{k} p^{ \frac{k(k-1)}{2}}}{q^{k} q^{ \frac{k(k-1)}{2}}} \frac{([n]_{p,q}x)^{k} }{ [k]_{p,q}! } \frac{[k+1]_{p,q}^{m} x}{p^{k(m+1)}[n]_{p,q}^{m}}e_{p,q} \bigl(-[n]_{p,q}q ^{-(k+1)}x \bigr) \\ ={}& \sum_{k=0}^{\infty} \frac{p^{k} p^{ \frac{k(k-1)}{2}}}{q^{k} q^{ \frac{k(k-1)}{2}}} \frac{([n]_{p,q}x)^{k} }{ [k]_{p,q}! } \frac{[k+1]_{p,q}^{m} x}{p^{km+k}[n]_{p,q}^{m}}e_{p,q} \bigl(-[n]_{p,q}q ^{-(k+1)}x \bigr) \\ ={}& \sum_{k=0}^{\infty} \frac{ p^{ \frac{k(k-1)}{2}}}{q^{k} q^{ \frac{k(k-1)}{2}}} \frac{([n]_{p,q}x)^{k} }{ [k]_{p,q}! } \frac{(p^{k}+q[k]_{p,q})^{m} x}{p^{km}[n]_{p,q}^{m}}e_{p,q} \bigl(-[n]_{p,q}q ^{-(k+1)}x \bigr) \\ ={}& \sum_{k=0}^{\infty} \frac{ x }{ p^{km} [n]_{p,q}^{m} } \frac{ p^{ \frac{k(k-1)}{2}}}{q^{k} q^{ \frac {k(k-1)}{2}}}\frac{([n]_{p,q}x)^{k} }{ [k]_{p,q}! }\\ &\times\sum_{j=0}^{m} \left ( \textstyle\begin{array}{c} m \\ j\end{array}\displaystyle \right )p^{k(m-j)}q^{j}[k]_{p,q}^{j}e_{p,q} \bigl(-[n]_{p,q}q ^{-(k+1)}x \bigr) \\ ={}& \sum_{j=0}^{m}\left ( \textstyle\begin{array}{c} m \\ j\end{array}\displaystyle \right ) \frac{ q^{j}x }{ p^{j} [n]_{p,q}^{m-j} }\\ &\times \sum _{k=0}^{\infty} \frac{ [k]_{p,q}^{j} }{ p^{j(k-1)} [n]_{p,q}^{j} } \frac{ p^{ \frac {k(k-1)}{2}}}{q^{k} q^{ \frac{k(k-1)}{2}}} \frac{([n]_{p,q}x)^{k} }{ [k]_{p,q}! } e_{p,q} \bigl(-[n]_{p,q}q ^{-(k+1)}x \bigr) \\ ={}& \sum_{j=0}^{m} \left ( \textstyle\begin{array}{c} m \\ j\end{array}\displaystyle \right ) \frac{q^{j}x }{p^{j} [n]_{p,q}^{m-j} } {S}_{n,p,q} \bigl(t^{j};q^{-1}x \bigr), \end{aligned} $$

as desired. □

### Lemma 2.2

*Let* $0< q< p\leq1$ *and* $n\in\mathbb{N}$. *We have*

(i) ${S}_{n,p,q}(1;x)=1$,
(ii) ${S}_{n,p,q}(t;x)=x$,
(iii) ${S}_{n,p,q}(t^{2};x)= \frac{x^{2}}{p}+\frac{ x}{[n]_{p,q} } $,
(iv) ${S}_{n,p,q}(t^{3};x)= \frac{x^{3}}{p^{3}}+ \frac{ 2p+q}{p^{2}[n]_{p,q} }x^{2} + \frac{ x}{[n]_{p,q}^{2} }$,
(v) ${S}_{n,p,q}(t^{4};x)= \frac{x^{4}}{p^{6}}+ \frac{3p^{2}+ 2pq+q^{2}}{p^{5}[n]_{p,q} }x^{3} + \frac{3p^{2}+ 3pq+q^{2}}{p^{3}[n]_{p,q}^{2} }x^{2}+ \frac{ x}{[n]_{p,q}^{3} }$.

### Proof

Since the proof of each equality uses the same method, we give the proof for only last three equalities. Using (2.2), we get

(iii) $$\begin{aligned} {S}_{n,p,q} \bigl(t^{2};x \bigr) ={}& \sum _{k=0}^{\infty} \frac{ p^{ \frac{k(k-1)}{2}}}{ q^{ \frac{k(k-1)}{2}}}\frac{([n]_{p,q}x)^{k}}{ [k]_{p,q}! } \frac {[k]_{p,q}^{2}}{p^{2k-2}[n]_{p,q}^{2}}e_{p,q} \bigl(-[n]_{p,q}q ^{-k}x \bigr) \\ ={}& \sum_{k=0}^{\infty} \frac{ p^{k}p^{ \frac{k(k-1)}{2}}}{ q^{k}q^{ \frac{k(k-1)}{2}}} \frac{([n]_{p,q}x)^{k}}{ [k]_{p,q}! } \frac{[k+1]_{p,q}x}{p^{2k}[n]_{p,q}}e_{p,q} \bigl(-[n]_{p,q}q ^{-(k+1)}x \bigr) \\ ={}& \sum_{k=0}^{\infty}\frac{ p^{k}p^{ \frac{k(k-1)}{2}}}{ q^{k}q^{ \frac{k(k-1)}{2}}} \frac{([n]_{p,q}x)^{k}}{ [k]_{p,q}! }\frac{p^{k}x}{p^{2k}[n]_{p,q}}e_{p,q} \bigl(-[n]_{p,q}q ^{-(k+1)}x \bigr) \\ &+ \sum_{k=0}^{\infty}\frac{ p^{ \frac{k(k-1)}{2}}}{ q^{k}q^{ \frac{k(k-1)}{2}}} \frac{([n]_{p,q}x)^{k}}{ [k]_{p,q}! }\frac{q [k]_{p,q}x }{p^{k}[n]_{p,q}}e_{p,q} \bigl(-[n]_{p,q}q ^{-(k+1)}x \bigr) \\ ={}& \frac{ x}{[n]_{p,q} }+ \sum_{k=0}^{\infty} \frac{ p^{ \frac{k(k-1)}{2}}}{ q^{2k}q^{ \frac {k(k-1)}{2}}}\frac{([n]_{p,q}x)^{k}}{ [k]_{p,q}! }\frac{x^{2} }{p} e_{p,q} \bigl(-[n]_{p,q}q ^{-(k+2)}x \bigr) \\ ={}& \frac{x^{2}}{p}+\frac{ x}{[n]_{p,q} }. \end{aligned}$$
(iv) $$\begin{aligned} {S}_{n,p,q} \bigl(t^{3};x \bigr) ={}& \sum _{k=0}^{\infty} \frac{ p^{ \frac{k(k-1)}{2}}}{ q^{ \frac{k(k-1)}{2}}}\frac{([n]_{p,q}x)^{k}}{ [k]_{p,q}! } \frac {[k]_{p,q}^{3}}{p^{3k-3}[n]_{p,q}^{3}}e_{p,q} \bigl(-[n]_{p,q}q ^{-k}x \bigr) \\ ={}& \sum_{k=0}^{\infty} \frac{ p^{ \frac{k(k-1)}{2}}}{ q^{k}q^{ \frac{k(k-1)}{2}}} \frac{([n]_{p,q}x)^{k}}{ [k]_{p,q}! } \frac {(p^{2k}+2p^{k}q[k]_{p,q}+q^{2}[k]_{p,q}^{2})}{p^{2k}[n]_{p,q}^{2}}\\ &\times x e_{p,q} \bigl(-[n]_{p,q}q ^{-(k+1)}x \bigr) \\ ={}& \sum_{k=0}^{\infty} \frac{ p^{ \frac{k(k-1)}{2}}}{q^{k} q^{ \frac{k(k-1)}{2}}} \frac{([n]_{p,q}x)^{k}}{ [k]_{p,q}! } \frac{x}{[n]_{p,q}^{2}}e_{p,q} \bigl(-[n]_{p,q}q ^{-(k+1)}x \bigr) \\ & +\sum_{k=0}^{\infty} \frac{ p^{ \frac{k(k-1)}{2}}}{ q^{k}q^{ \frac{k(k-1)}{2}}} \frac{([n]_{p,q}x)^{k}}{ [k]_{p,q}! } \frac{2q[k]_{p,q}}{p^{k}[n]_{p,q}^{2}}x e_{p,q} \bigl(-[n]_{p,q}q ^{-(k+1)}x \bigr) \\ &+ \sum_{k=0}^{\infty} \frac{ p^{ \frac{k(k-1)}{2}}}{ q^{k}q^{ \frac{k(k-1)}{2}}} \frac{([n]_{p,q}x)^{k}}{ [k]_{p,q}! } \frac{q^{2}[k]_{p,q}^{2}}{p^{2k}[n]_{p,q}^{2}}x e_{p,q} \bigl(-[n]_{p,q}q ^{-(k+1)}x \bigr) \\ ={}& \frac{ x}{[n]_{p,q}^{2} } +\frac{ 2x^{2}}{p[n]_{p,q} }\\ &+ \sum_{k=0}^{\infty} \frac{p^{k} p^{ \frac{k(k-1)}{2}}}{ q^{2k}q^{ \frac{k(k-1)}{2}}}\frac{([n]_{p,q}x)^{k}}{ [k]_{p,q}! } \frac{q x^{2} (p^{k}+ q[k]_{p,q})}{p^{2k+2}[n]_{p,q}} e_{p,q} \bigl(-[n]_{p,q}q ^{-(k+2)}x \bigr) \\ ={}& \frac{ x}{[n]_{p,q}^{2} } +\frac{ 2x^{2}}{p[n]_{p,q} }+ \frac{ qx^{2}}{p^{2}[n]_{p,q} } \\ &+ \sum _{k=0}^{\infty} \frac{ p^{ \frac{k(k-1)}{2}}}{ q^{2k}q^{ \frac{k(k-1)}{2}}}\frac{([n]_{p,q}x)^{k}}{ [k]_{p,q}! } \frac{q^{2} x^{2} [k]_{p,q}}{p^{k+2}[n]_{p,q}} e_{p,q} \bigl(-[n]_{p,q}q ^{-(k+2)}x \bigr) \\ ={}& \frac{x^{3}}{p^{3}}+ \frac{ 2p+q}{p^{2}[n]_{p,q} }x^{2} + \frac{ x}{[n]_{p,q}^{2} }. \end{aligned}$$
(v) $$\begin{aligned} {S}_{n,p,q} \bigl(t^{4};x \bigr) ={}& \sum _{k=0}^{\infty} \frac{ p^{ \frac{k(k-1)}{2}}}{ q^{ \frac{k(k-1)}{2}}}\frac{([n]_{p,q}x)^{k}}{ [k]_{p,q}! } \frac {[k]_{p,q}^{4}}{p^{4k-4}[n]_{p,q}^{4}}e_{p,q} \bigl(-[n]_{p,q}q ^{-k}x \bigr) \\ ={}& \sum_{k=0}^{\infty} \frac{ p^{ \frac{k(k-1)}{2}}}{ q^{k}q^{ \frac{k(k-1)}{2}}} \frac{([n]_{p,q}x)^{k}}{ [k]_{p,q}! } \frac{(p^{3k}+3p^{2k}q[k]_{p,q}+3p^{k}q^{2}[k]_{p,q}^{2}+q^{3}[k]_{p,q}^{3})}{ p^{3k}[n]_{p,q}^{3}}\\ &\times x e_{p,q} \bigl(-[n]_{p,q}q ^{-(k+1)}x \bigr) \\ ={}& \frac{ x}{[n]_{p,q}^{3} } +\frac{ 3x^{2}}{p[n]_{p,q}^{2} }+ \frac{ 3qx^{2}}{p^{2}[n]_{p,q}^{2} }+ \frac{ 3x^{3}}{p^{3}[n]_{p,q} } \\ &+ \sum_{k=0}^{\infty} \frac{ p^{ \frac{k(k-1)}{2}}}{ q^{2k}q^{ \frac{k(k-1)}{2}}} \frac{([n]_{p,q}x)^{k}}{ [k]_{p,q}! } \frac{q^{2} x^{2} (p^{2k}+ 2p^{k}q[k]_{p,q}+q^{2} [k]_{p,q}^{2} )}{p^{2k+3}[n]_{p,q}^{2}}\\ &\times e_{p,q} \bigl(-[n]_{p,q}q ^{-(k+2)}x \bigr) \\ ={}& \frac{ x}{[n]_{p,q}^{3} } +\frac{ 3x^{2}}{p[n]_{p,q}^{2} }+ \frac{ 3qx^{2}}{p^{2}[n]_{p,q}^{2} }+ \frac{ 3x^{3}}{p^{3}[n]_{p,q} }+\frac{ q^{2}x^{2}}{p^{3}[n]_{p,q}^{2} } + \frac{ 2qx^{3}}{p^{4}[n]_{p,q} } \\ &+ \sum_{k=0}^{\infty} \frac{ p^{ \frac{k(k-1)}{2}}}{ q^{3k}q^{ \frac{k(k-1)}{2}}} \frac{([n]_{p,q}x)^{k}}{ [k]_{p,q}! } \frac{q^{2} x^{3} (p^{k}+ q[k]_{p,q} )}{p^{k+5}[n]_{p,q}} e_{p,q} \bigl(-[n]_{p,q}q ^{-(k+3)}x \bigr) \\ ={}& \frac{x^{4}}{p^{6}}+ \frac{3p^{2}+ 2pq+q^{2}}{p^{5}[n]_{p,q} }x^{3} + \frac{3p^{2}+ 3pq+q^{2}}{p^{3}[n]_{p,q}^{2} }x^{2}+ \frac{ x}{[n]_{p,q}^{3} }. \end{aligned}$$

 □

### Corollary 2.1

*Using Lemma* 2.2, *we immediately have the following explicit formulas for the central moments*:

2.3 $$\begin{aligned}& {S}_{n,p,q} \bigl((t-x)^{2};x \bigr) = \frac{ x}{[n]_{p,q} }+ \biggl(\frac{1}{p}-1 \biggr)x^{2}, \end{aligned}$$

2.4 $$\begin{aligned}& {S}_{n,p,q} \bigl((t-x)^{3};x \bigr)= \frac{ x}{[n]_{p,q}^{2} }+ \frac {2p+q-3p^{2}}{p^{2}[n]_{p,q}} x^{2} +\frac{1-3p^{2}+2p^{3}}{p^{3}} x^{3}, \end{aligned}$$

2.5 $$\begin{aligned}& \begin{aligned}[b] {S}_{n,p,q} \bigl((t-x)^{4};x \bigr) ={}& \frac{ x}{[n]_{p,q}^{3} }+ \frac{3p^{2}+3pq+ q^{2}-4p^{3}}{p^{3}[n]_{p,q}^{2}} x^{2} \\ &+\frac{3p^{2}+2pq+ q^{2}-8p^{4}-4p^{3}q+6p^{5}}{p^{5}[n]_{p,q}} x^{3}\\ &+ \frac{1-4p^{3}+6p^{5}-3p^{6}}{p^{6}}x^{4}. \end{aligned} \end{aligned}$$

### Remark 2.1

For $q\in(0, 1)$ and $p\in(q, 1]$ we easily see that $\lim_{n\rightarrow\infty}[n]_{p,q}=\frac{1}{p-q}$. Hence, operators (2.1) are not approximation process with above form. To study convergence properties of the sequence of $(p, q)$-Szász operators, we assume that $q = (q_{n})$ and $p = (p_{n})$ are such that $0 < q_{n} < p_{n} \leq1$ and $q_{n} \rightarrow1$, $p_{n} \rightarrow1$, $q_{n} ^{n} \rightarrow a$, $p^{n}_{n} \rightarrow b$ as $n \rightarrow\infty$. We also assume that

$$ \begin{gathered} \lim_{n\rightarrow\infty}[n]_{p_{n},q_{n}} \biggl( \frac{1}{p_{n}}-1 \biggr) = \alpha, \\ \lim_{n\rightarrow\infty}[n]_{p_{n},q_{n}} \frac {1-3p^{2}_{n}+2p^{3}_{n}}{p^{3}_{n}} = \gamma, \\ \lim_{n\rightarrow\infty}[n]_{p_{n},q_{n}} \frac{1-4p^{3}_{n}+6p^{5}_{n}-3p^{6}_{n}}{p^{6}_{n}} =\beta. \end{gathered} $$

It is natural to ask whether such sequences $(q_{n})$ and $(p_{n})$ exist. For example, let $c, d \in\mathbb{R^{+}}$ be such that $c > d$. If we choose $q_{n}=\frac{n}{n+c}$ and $p_{n}=\frac{n}{n+d}$, then $q_{n} \rightarrow1$, $p_{n} \rightarrow1$, $q^{n}_{n} \rightarrow e^{-c}$, $p^{n}_{n} \rightarrow e^{-d}$, and $\lim_{n\rightarrow\infty}[n]_{p,q}=\infty$ as $n \rightarrow \infty$. Also, we have $\alpha=\frac{a(e^{-d}- e^{-c}) }{d-c}$, $\gamma=e^{-d}- e^{-c}$, $\beta=0$.

### Corollary 2.2

*According to Remark* 2.1, *we immediately have*

2.6 $$\begin{aligned}& \lim_{n\rightarrow\infty}[n]_{p_{n},q_{n}}{S}_{n,p_{n},q_{n}} \bigl((t-x)^{2};x \bigr) = x+\alpha x^{2}, \end{aligned}$$

2.7 $$\begin{aligned}& \lim_{n\rightarrow\infty}[n]_{p_{n},q_{n}}{S}_{n,p_{n},q_{n}} \bigl((t-x)^{3};x \bigr)= \gamma x^{3}, \end{aligned}$$

2.8 $$\begin{aligned}& \lim_{n\rightarrow\infty}[n]_{p_{n},q_{n}}{S}_{n,p_{n},q_{n}} \bigl((t-x)^{4};x \bigr) = \beta x^{4}. \end{aligned}$$

## Direct results

In this section, we present a local approximation theorem for the operators $S_{n,p,q}$. By $C_{B}[0,\infty)$ we denote the space of real-valued continuous and bounded functions *f* defined on the interval $[0,\infty)$. The norm $\|\cdot\|$ on the space $C_{B}[0,\infty)$ is given by

$$ \| f\|=\sup_{0\leq x< \infty}\big| f(x)\big|. $$

Further, let us consider the following *K*-functional:

$$ K_{2}(f,\delta)=\inf_{g\in W^{2}} \bigl\{ \| f-g \|+\delta \big\| g^{{\prime\prime}}\big\| \bigr\} , $$

where $\delta>0$ and $W^{2}=\{g\in C_{B}[0,\infty):g^{{\prime}},g^{{\prime\prime}}\in C_{B}[0,\infty)\}$. By Theorem 2.4 of [22] there exists an absolute constant $C>0$ such that

3.1 $$ K_{2}(f,\delta)\leq C\omega_{2}(f,\sqrt{\delta}), $$

where

$$ \omega_{2} (f,\sqrt{\delta})=\sup_{0< h\leq\sqrt{\delta}} \sup _{x\in [0,\infty)}\big| f(x+2h)-2f(x+h)+f(x)\big| $$

is the second-order modulus of smoothness of $f\in C_{B} [0,\infty)$. The usual modulus of continuity of $f\in C_{B} [0,\infty)$ is defined by

$$ \omega(f,\delta)=\sup_{0< h\leq\delta} \sup_{x\in[0,\infty)}\big| f(x+h)-f(x)\big|. $$

### Theorem 3.1

*Let* $p,q \in(0,1)$ *be such that* $q < p$. *Then we have*

$$ \big|{S}_{n,p,q}(f;x)-f(x)\big|\leq C \omega_{2} \bigl(f; \delta_{n}(x) \bigr) $$

*for every* $x\in[0,\infty)$ *and* $f\in C_{B} [0,\infty)$, *where*

$$ \delta_{n}^{2}(x)=\frac{ x}{[n]_{p,q} }+ \biggl( \frac{1}{p}-1 \biggr)x^{2}. $$

### Proof

Let $g\in\mathcal{W}^{2}$. Then from the Taylor expansion we get

$$ g(t)=g(x)+g^{\prime}(x) (t-x)+ \int_{x}^{t}(t-u)g^{\prime\prime}(u) \,\mathrm{d}u, \quad t\in[0,\mathcal{A}], \mathcal{A}>0. $$

Now by Corollary 2.1 we have

$$\begin{gathered} {S}_{n,p,q}(g;x)=g(x)+{S}_{n,p,q} \biggl( \int_{x}^{t}(t-u)g^{\prime\prime}(u)\,\mathrm{d}u;x \biggr), \\\begin{aligned}{\big|} {S}_{n,p,q}(g;x)-g(x) {\big|} &\leq{S}_{n,p,q} \biggl( {\bigg|}\int_{x}^{t}\big|(t-u)\big| \big| g^{\prime\prime}(u) \big| \mathrm{d}u;x {\bigg|} \biggr) \\ &\leq{S}_{n,p,q} \bigl( (t-x)^{2};x \bigr) \big\| g^{\prime\prime} \big\| . \end{aligned}\end{gathered}$$

Hence we get

$$ {\big|} {S}_{n,p,q}(g;x)-g(x) {\big|}\leq\big\| g^{\prime\prime}\big\| \biggl( \frac{x}{[n]_{p,q}}+ \biggl(\frac{1}{p}-1 \biggr)x^{2} \biggr) . $$

On the other hand, we have

$$ {\big|} {S}_{n,p,q}(f;x)-f(x) {\big|} \leq \big|{S}_{n,p,q} \bigl( (f-g);x \bigr) -(f-g) (x)\big|+ {\big|} {S}_{n,p,q}(g;x)-g(x) {\big|}. $$

Since

$$ \big|{S}_{n,p,q}(f;x)\big|\leq\| f\|, $$

we have

$$ {\big|} {S}_{n,p,q}(f;x)-f(x) {\big|} \leq \| f-g\| +\big\| g^{\prime\prime}\big\| \biggl( \frac{x}{[n]_{p,q}}+ \biggl(\frac {1}{p}-1 \biggr)x^{2} \biggr) . $$

Now taking the infimum on the right-hand side over all $g\in\mathcal {W}^{2}$, we get

$$ {\big|} {S}_{n,p,q}(f;x)-f(x) {\big|} \leq \mathcal{C}K_{2} \bigl( f, \delta_{n}^{2}(x) \bigr) . $$

By the property of a *K*-functional we get

$$ {\big|} {S}_{n,p,q}(f;x)-f(x) {\big|} \leq \mathcal{C}\omega _{2} \bigl( f,\delta_{n}(x) \bigr) . $$

This completes the proof. □

## Weighted approximation by $S_{n,p,q}$

Now we give approximation properties of the operators $S_{n,p,q}$ on the interval $[0,\infty)$. Since

$$\begin{aligned} S_{n,p,q} \bigl(1+t^{2};x \bigr) &=1+ \biggl( \frac{1}{p}-1 \biggr)x^{2}+\frac{x}{[n]_{p,q}} \\ &\leq1+x^{2}+x, \end{aligned}$$

$x\leq1$ for $x\in{}[0,1]$, and $x\leq x^{2}$ for $x\in(1,\infty)$, we have

$$ S_{n,p,q} \bigl(1+t^{2};x \bigr)\leq2 \bigl(1+x^{2} \bigr), $$

which says that $S_{n,p,q}$ are linear positive operators acting from $C_{2}[0,\infty)$ to $B_{2}[0,\infty)$. For more details, see [23, 24], and [25].

### Theorem 4.1

*Let the sequence of linear positive operators* $(L_{n})$ *acting from* $C_{2} [0,\infty)$ *to* $B_{2} [0,\infty)$ *satisfy the condition*

$$ \lim_{n\rightarrow\infty}\| L_{n}e_{i}-e_{i} \|_{2}=0 ,\quad i=0, 1, 2. $$

*Then*, *for any function* $f \in C_{2}^{\ast} [0,\infty) $,

$$ \lim_{n\rightarrow\infty}\| L_{n}f-f\|_{2}=0. $$

### Theorem 4.2

*Let* $q = q_{n}\in(0, 1)$ *and* $p = p_{n}\in(q, 1)$ *be such that* $q_{n}\rightarrow1$ *and* $p_{n}\rightarrow1$ *as* $n \rightarrow\infty$. *Then*, *for each function* $f \in C_{2}^{\ast} [0,\infty)$, *we get*

$$ \lim_{n\rightarrow\infty}\| S_{n,p_{n},q_{n}}f-f\|_{2}=0. $$

### Proof

According to Theorem 4.1, it is sufficient to verify the condition

4.1 $$ \lim_{n\rightarrow\infty}\| S_{n,p_{n},q_{n}}e_{i}-e_{i} \|_{2} = 0,\quad i=0, 1, 2. $$

By Lemma 2.1(i), (ii) it is clear that

$$\begin{gathered} \lim_{n\rightarrow\infty}\big\| S_{n,p_{n},q_{n}}(1;x)-1\big\| _{2} =0, \\ \lim_{n\rightarrow\infty}\big\| S_{n,p_{n},q_{n}}(t;x)-x \big\| _{2} =0, \end{gathered}$$

and by Lemma 2.1(iii) we have

$$\begin{aligned} \lim_{n\rightarrow\infty}\big\| S_{n,p_{n},q_{n}} \bigl(t^{2};x \bigr)-x^{2}\big\| _{2} &=\sup_{x\geq0} \frac {(\frac{1}{p_{n}}-1)x^{2}+\frac{x}{[n]_{p_{n},q_{n}}}}{1+x^{2}} \\ &\leq \biggl(\frac{1}{p_{n}}-1 \biggr)+\frac{1}{[n]_{p_{n},q_{n}}}. \end{aligned}$$

The last inequality means that (4.1) holds for $i=2$. By Theorem 4.1 the proof is complete. □

The weighted modulus of continuity is given by

4.2 $$ \Omega(f; \delta) = \sup_{0\leq h < \delta,x\in[0, \infty) } \frac {| f(x+h)-f(x)|}{(1+h^{2})+(1+x^{2})} $$

for $f \in C_{2} [0,\infty)$. We know that, for every $f \in C_{2}^{\ast} [0,\infty)$, $\Omega(\cdot; \delta)$ has the properties

$$ \lim_{\delta\rightarrow0}\Omega(f; \delta)=0 $$

and

4.3 $$ \Omega(f; \lambda\delta) \leq 2(1+\lambda) \bigl(1+ \delta^{2} \bigr) \Omega(f; \delta), \quad\lambda>0. $$

For $f \in C_{2} [0,\infty)$, from (4.2) and (4.3) we can write

4.4 $$ \begin{aligned}[b] \big| f(t)-f(x)\big|&\leq \bigl(1+(t-x)^{2} \bigr) \bigl(1+x^{2} \bigr)\Omega\bigl(f; | t-x|\bigr) \\ &\leq 2 \biggl(1+\frac{| t-x|}{\delta} \biggr) \bigl(1+\delta^{2} \bigr) \Omega(f; \delta) \bigl(1+(t-x)^{2} \bigr) \bigl(1+x^{2} \bigr). \end{aligned} $$

All concepts mentioned can be found in [26].

### Theorem 4.3

*Let* $0< q = q_{n} < p = p_{n}\leq1$ *be such that* $q_{n}\rightarrow 1$ *and* $p_{n}\rightarrow1$ *as* $n \rightarrow\infty$. *Then*, *for each function* $f \in C_{2}^{\ast} [0,\infty)$, *there exists a positive constant A such that*

$$ \sup_{x\in[0, \infty) } \frac{| S_{n,p,q}(f;x)-f(x)| }{(1+x^{2})^{\frac{5}{2}}} \leq A\Omega \biggl(f; \frac{1}{ \sqrt{\beta_{p,q}(n)}} \biggr), $$

*where* $\beta_{p,q}(n)= \max \{\frac{1}{p}-1 ,\frac{1}{[n]_{p,q}} \}$, *and A is a positive constant*.

### Proof

Since $S_{n,p,q}(1; x) = 1$, using the monotonicity of $S_{n,p,q}$, we can write

$$ \big| S_{n,p,q}(f;x)-f(x)\big|\leq S_{n,p,q} \bigl(\big| f(t)-f(x) \big|;x \bigr). $$

On the other hand, from (4.4) we have that

$$\begin{aligned} \big| S_{n,p,q}(f;x)-f(x)\big|\leq{}& 2 \bigl(1+\delta^{2} \bigr) \Omega(f; \delta) \bigl(1+x^{2} \bigr) \biggl[S_{n,p,q} \biggl( \biggl(1+\frac{| t-x| }{\delta} \biggr) \bigl(1+(t-x)^{2} \bigr);x \biggr) \biggr] \\ \leq{}& 2 \bigl(1+\delta^{2} \bigr)\Omega(f; \delta) \bigl(1+x^{2} \bigr) \biggl\{ S_{n,p,q}(1;x)+S_{n,p,q} \bigl((t-x)^{2};x \bigr) \\ &+\frac{1}{\delta}S_{n,p,q}\bigl(| t-x|;x \bigr)+\frac{1}{\delta}S_{n,p,q} \bigl(| t-x|(t-x)^{2} ;x \bigr) \biggr\} . \end{aligned}$$

Using the Cauchy-Schwarz inequality, we can write

$$\begin{aligned} \big| S_{n,p,q}(f;x)-f(x)\big|\leq{}& 2 \bigl(1+\delta^{2} \bigr) \Omega(f; \delta) \bigl(1+x^{2} \bigr) \biggl\{ S_{n,p,q}(1;x)+S_{n,p,q} \bigl((t-x)^{2};x \bigr) \\ &+ \frac{1}{\delta} \sqrt{S_{n,p,q} \bigl((t-x)^{2} ;x \bigr)} +\frac{1}{\delta }\sqrt{S_{n,p,q} \bigl( (t-x)^{4} ;x \bigr)} \sqrt{S_{n,p,q} \bigl( (t-x)^{2} ;x \bigr)} \biggr\} . \end{aligned}$$

On the other hand, using (2.3), we have

$$\begin{aligned} S_{n,p,q} \bigl((t-x)^{2} ;x \bigr) &\leq \frac{ x}{[n]_{p,q} }+ \biggl(\frac {1}{p}-1 \biggr)x^{2} \\ &\leq C_{1}O \bigl(\beta_{p,q}(n) \bigr) \bigl(1+x^{2} \bigr), \end{aligned}$$

where $C_{1} > 0$ and $\beta_{p,q}(n)= \max \{\frac{1}{p}-1 ,\frac {1}{[n]_{p,q}} \}$. Since $\lim_{n\rightarrow\infty}\frac {1}{p_{n}}=1$ and $\lim_{n\rightarrow\infty}\frac{1}{[n]_{p,q}}=0$, there exists a positive constant $A_{2}$ such that

$$ S_{n,p,q} \bigl((t-x)^{2} ;x \bigr) \leq A_{2} \bigl(1+x^{2} \bigr). $$

Also, using (2.5), we get

$$ S_{n,p,q} \bigl( (t-x)^{4} ;x \bigr)^{\frac{1}{2}} \leq A_{3} \bigl(1+x^{2} \bigr) $$

and

$$ S_{n,p,q} \biggl( \frac{(t-x)^{2}}{\delta^{2}} ;x \biggr)^{\frac{1}{2}} \leq \frac{A_{4}}{\delta} O \bigl(\beta_{p,q}(n) \bigr)^{\frac{1}{2}} \bigl(1+x^{2} \bigr)^{\frac{1}{2}} $$

for $A_{3} > 0$ and $A_{4} > 0$. So we have

$$\begin{aligned} \big| S_{n,p,q}(f;x)-f(x)\big|\leq{}& 2 \biggl(1+\frac{1}{\beta_{p,q}(n)} \biggr) \Omega \biggl(f; \frac{1}{\sqrt{\beta_{p,q}(n)}} \biggr) \bigl(1+x^{2} \bigr) \biggl\{ 1+ A_{2} \bigl(1+x^{2} \bigr) \\ &+ \frac{A_{4}}{\delta} O \bigl(\beta_{p,q}(n) \bigr)^{\frac{1}{2}} \bigl(1+x^{2} \bigr)^{\frac{1}{2}} \\ &+A_{3} \bigl(1+x^{2} \bigr)\frac{A_{4}}{\delta}O \bigl(\beta_{p,q}(n) \bigr)^{\frac{1}{2}} \bigl(1+x^{2} \bigr)^{\frac{1}{2}} \biggr\} . \end{aligned}$$

Choosing $\delta= \beta_{p,q}(n)^{\frac{1}{2}}$, we obtain

$$\begin{aligned} \big| S_{n,p,q}(f;x)-f(x)\big|\leq{}& 2 \bigl(1+\beta_{p,q}(n) \bigr) \Omega \biggl(f; \frac {1}{\sqrt{\beta_{p,q}(n)}} \biggr) \bigl(1+x^{2} \bigr) \bigl\{ 1+ A_{2} \bigl(1+x^{2} \bigr) \\ &+ CA_{4} \bigl(1+x^{2} \bigr)^{\frac{1}{2}} +C_{1}A_{3}A_{4} \bigl(1+x^{2} \bigr)^{\frac {3}{2}} \bigr\} . \end{aligned}$$

For $0 < q < p \leq1$, we have $\beta_{p,q}(n) \leq1$. Hence we can write

$$ \sup_{x\in[0, \infty) } \frac{| S_{n,p,q}(f;x)-f(x)| }{(1+x^{2})^{\frac{5}{2}}} \leq A\Omega \biggl(f; \frac{1}{ \sqrt{\beta_{p,q}(n)}} \biggr), $$

where $A = 4 (1 + A_{2} + CA_{4} + C_{1}A_{3}A_{4})$, and the result follows. □

## Voronovskaya-type theorem for $S_{n,p,q}$

Here we give a Voronovskaya-type theorem for $S_{n,p,q}$.

### Theorem 5.1

*Let* $0< q_{n} < p_{n}\leq1$ *be such that* $q_{n}\rightarrow 1$, $p_{n}\rightarrow1$, $q_{n}^{n}\rightarrow a$, *and* $p_{n}^{n}\rightarrow b$ *as* $n \rightarrow\infty$. *Then*, *for each function* $f \in C_{2}^{\ast} [0,\infty)$ *such that* $f^{{\prime}},f^{{\prime\prime}} \in C_{2}^{\ast} [0,\infty)$, *we have*

$$ \lim_{n\rightarrow\infty} [n]_{p_{n},q_{n}} \bigl( S_{n,p_{n},q_{n}}(f;x)-f(x) \bigr)= \bigl(x+\alpha x^{2} \bigr)f^{{\prime\prime}}(x) $$

*uniformly on any* $[0,A] $, $A > 0$.

### Proof

Let $f,f^{{\prime}},f^{{\prime\prime}} \in C_{2}^{\ast} [0,\infty )$ and $x \in[0,\infty)$. By the Taylor formula we can write

5.1 $$ f(t) = f(x)+f^{{\prime}}(x) (t-x)+\frac{1}{2}f^{{\prime\prime }}(x) (t-x)^{2}+h(t,x) (t-x)^{2}, $$

where $h (t, x)$ is the remainder of the Peano form. Then $h (\cdot, x) \in C_{2}^{\ast} [0,\infty)$ and $\lim_{t\rightarrow x}h (t, x)=0$ for *n* large enough. Applying operators (2.1) to both sides of (5.1), we get

$$\begin{aligned} [n]_{p_{n},q_{n}} \bigl( S_{n,p_{n},q_{n}}(f;x)-f(x) \bigr)={}&[n]_{p_{n},q_{n}}f^{{\prime }}(x)S_{n,p_{n},q_{n}} \bigl((t-x);x \bigr)\\ & + [n]_{p_{n},q_{n}}f^{{\prime \prime}}(x)S_{n,p_{n},q_{n}} \bigl((t-x)^{2};x \bigr) \\ &+S_{n,p_{n},q_{n}} \bigl(h (t, x) (t-x)^{2};x \bigr). \end{aligned}$$

By the Cauchy-Schwarz inequality we have

5.2 $$ S_{n,p_{n},q_{n}} \bigl(h (t, x) (t-x)^{2};x \bigr) \leq \sqrt{S_{n,p_{n},q_{n}} \bigl(h^{2} (t, x);x \bigr)} \sqrt{S_{n,p_{n},q_{n}} \bigl((t-x)^{4};x \bigr)} . $$

Observe that $h^{2} (x, x) = 0$ and $h^{2} (\cdot, x)\in C_{2}^{\ast} [0,\infty)$. Then it follows from Theorem 4.3 that

5.3 $$ \lim_{n\rightarrow\infty} S_{n,p_{n},q_{n}} \bigl(h^{2} (t, x);x \bigr) = h^{2} (x, x)=0 $$

uniformly with respect to $x \in[0,A]$. Hence, from (5.2), (5.3), and (2.8) we obtain

5.4 $$ \lim_{n\rightarrow\infty}[n]_{p_{n},q_{n}} S_{n,p_{n},q_{n}} \bigl(h (t, x) (t-x)^{2};x \bigr) = 0 $$

and

$$ S_{n,p,q} \bigl((t-x);x \bigr) = 0. $$

Then using (2.6) and (5.4), we have

$$\begin{aligned} \lim_{n\rightarrow\infty} [n]_{p_{n},q_{n}} \bigl( S_{n,p_{n},q_{n}}(f;x)-f(x) \bigr) ={}&f^{{\prime }}(x)\lim_{n\rightarrow\infty} [n]_{p_{n},q_{n}}S_{n,p_{n},q_{n}} \bigl((t-x);x \bigr) \\ &+ f^{{\prime\prime}}(x)\lim_{n\rightarrow\infty} [n]_{p_{n},q_{n}}S_{n,p_{n},q_{n}} \bigl((t-x)^{2};x \bigr) \\ &+ \lim_{n\rightarrow\infty}[n]_{p_{n},q_{n}}S_{n,p_{n},q_{n}} \bigl(h (t, x) (t-x)^{2};x \bigr) \\ ={}& \bigl(x+\alpha x^{2} \bigr)f^{{\prime\prime}}(x), \end{aligned}$$

as desired. □

## Conclusion

In this paper, we have constructed a new modification of Szász-Mirakyan operators based on $(p,q)$-integers and investigated their approximation properties. We have obtained a weighted approximation and Voronovskaya-type theorem for our new operators.
